# The Fire Service for the Removal of a Metallic Penile Constricting Device: A Ready Help When All Else Fail

**DOI:** 10.1155/2018/7543451

**Published:** 2018-09-25

**Authors:** Olufunmilade A. Omisanjo, Olufemi Ojewuyi, Abimbola Abolarinwa, Oluwaseun Akinola, Mofeyisayo Omorinde, Olabode Oshodi, Stephen Ikuerowo, Ayoade Adedokun, David Oke

**Affiliations:** ^1^Department of Surgery, Lagos State University College of Medicine and Teaching Hospital (LASUCOM/LASUTH), Ikeja, Lagos, Nigeria; ^2^Department of Surgery, Lagos State University Teaching Hospital (LASUTH), Ikeja, Lagos, Nigeria; ^3^General Outpatient Department, Lagos State University Teaching Hospital (LASUTH), Ikeja, Lagos, Nigeria; ^4^Department of Medicine, Lagos State University Teaching Hospital (LASUTH), Ikeja, Lagos, Nigeria

## Abstract

The placement of a constricting device around the penis is a urologic emergency. Though injuries from constricting penile devices are generally rare, they may be associated with serious complications. There is no standard modality for the removal of penile constricting devices and the management of the patient can therefore prove to be a formidable challenge to the urologist. Timely intervention is always important in preventing complications especially penile gangrene. Depending on the type of device used along with the duration and severity of penile constriction caused, significant resourcefulness may be required in the treatment of the patient. Achieving a timely and successful outcome may require a multidisciplinary approach involving equipment only available with the fire service or other agencies. We report the case of a 30-year-old man with a background psychiatric illness who had his penile constricting device removed under conscious sedation in the emergency room with the aid of a power driven arc saw from the fire service with a successful outcome.

## 1. Introduction

Penile constriction injury is a urologic emergency. It can lead to impaired venous and lymphatic drainage with the resultant increase in hydrostatic pressure leading to loss of fluid into the interstitial tissue and penile oedema [1]. If the penile constriction remains unresolved, ischemia results and may progress to a gangrenous penis. Emergency treatment requires decompression of the constricted penis to improve arterial inflow and venous return.

There are different forms of penile constricting devices reported in the literature ranging from the use of hair strands, strings, rubber bands, plastic bottles, beer bottles to PVC pipes, and metal rings [2–6]. Depending on the type of device used along with the duration and severity of penile constriction caused, significant resourcefulness and a multidisciplinary approach may be required in the treatment of the patient.

Numerous methods have been described in the literature for the removal of penile constricting devices. These include lubrication of the penile shaft and attempt at removal of the device, the string method, penile degloving, and the use of industrial drills, steel saws, hacksaws, and high-speed electric drills [4, 7, 8]. The most common method of removal is usually some sort of cutting of the device [9]. Rarely, fire service equipment may be required to cut through iron and steel rings [10].

We report the use of a Fire Department power driven arc saw in the removal of a very thick metallic penile constricting ring in a 30-year-old Nigerian male at the Lagos State University Teaching Hospital Ikeja Lagos Nigeria.

## 2. Case Report

We present the case of a 30-year-old Nigerian male who was brought to the Surgical Emergency Department of the Lagos State University Teaching Hospital Ikeja 22 hours after he had inserted a constricting ring over his penis. He had developed a painful penile shaft swelling distal to the ring with suprapubic pain and swelling secondary to acute urinary retention. There was associated urethral bleeding.

There had been failed attempts at removing the ring by self and the resulting severe pain drew the attention of his relatives who brought him to the emergency room.

He had a history of a psychiatric illness and the patient claimed he was under a spell and had heard a voice that instructed him to insert a ring over his penis. He denied using the ring to sustain erection and claimed it was his first time of inserting a ring over his penis.

The patient had a history of deterioration in personal and general performance with underachievement dating back to 7 years prior to presentation when he voluntarily dropped out of the university and had done nothing tangible thereafter.

Two weeks prior to presentation, the patient's relatives had noticed some unusual behavior in him characterized by talking to self and rubbing salt over his body and the patient claimed he was being chased by unseen people.

He had a history of alcohol, cigarette, and cannabis abuse for about 15 years.

On examination, he was in acute urinary retention with a tender suprapubic distention up to the level of the umbilicus.

**Figure 1:**
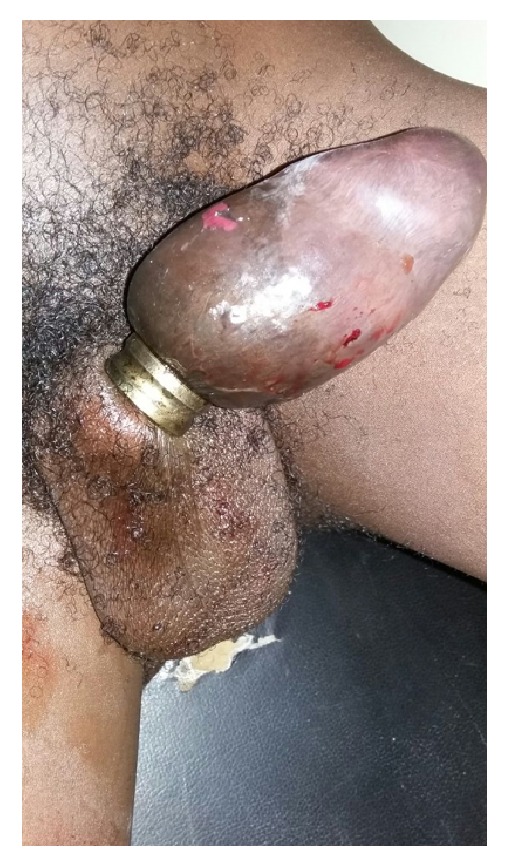
Thick constricting penile ring with grossly swollen penis with skin changes.

There was a thick constricting ring at the root of his penis. There was a markedly swollen oedematous penis distal to the ring with marked reduction in sensation over the penis and glans (Figure 1).

We made a diagnosis of Constrictive Penile Injury (Bhat Grade III) with acute urinary retention [11].

He had a suprapubic cystostomy done to relieve the acute urinary retention as a urethral catheterization was impossible.

Attempts were made to remove the constricting ring by the use of aspiration, application of cold compress, and lubrication initially and later by the use of the string method.

Following failed attempts at removing the device with these different manipulations and unsuccessful attempt at cutting with the manual saws available in the hospital coupled with the fact that the patient appeared to have imminent penile gangrene, a decision was made to call the fire service for a power driven saw.

**Figure 2:**
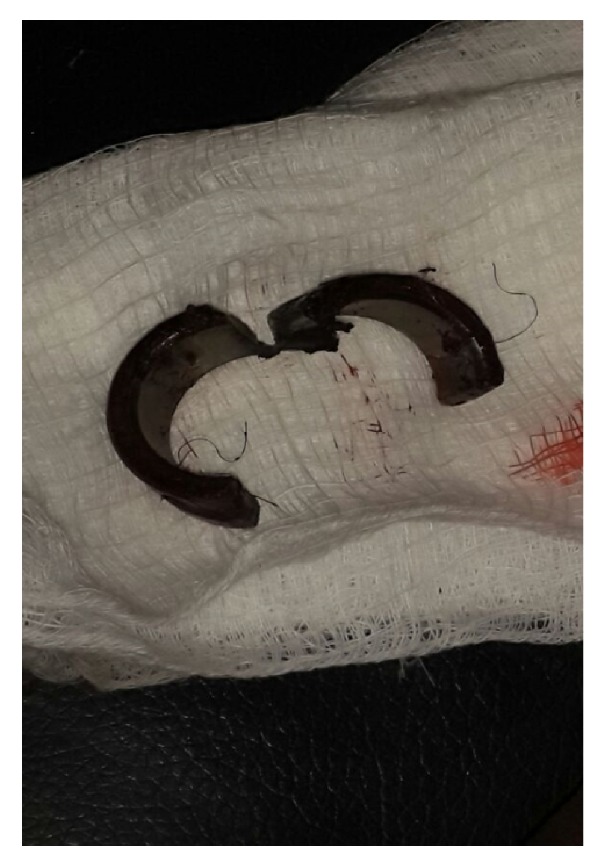
Thick constricting penile ring cut in 2 places.

**Figure 3:**
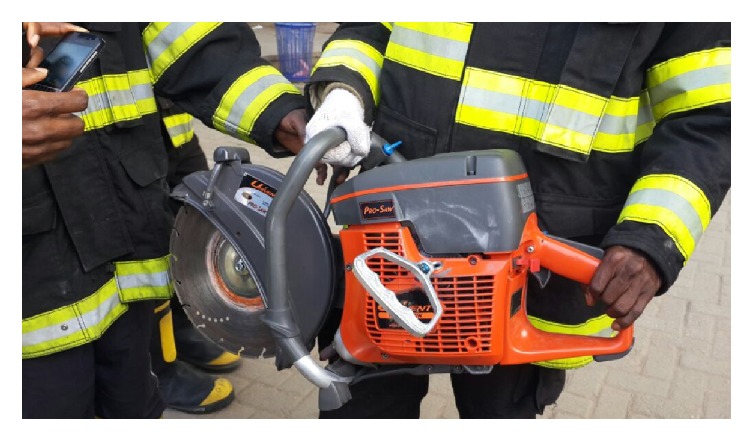
Power driven arc saw used by the men of the Lagos State Fire Service.

The ring was successfully removed by cutting it at two different points (Figure 2) with a power driven arc saw (Figure 3) under conscious sedation at the emergency room.

**Figure 4:**
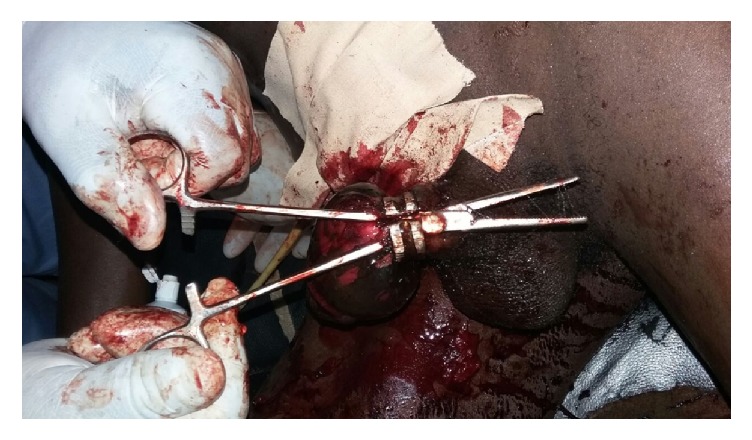
Artery Forceps insinuated between the penis and the ring to minimize risk of penile damage while cutting the ring.

Thermal injury was prevented by intermittent cooling with ice packs and injury to underlying tissue was prevented by insinuating a pair of artery forceps between the penis and the ring (Figure 4).

Dressing of the resulting penile skin ulceration was done and the plastic surgery team was invited for possible additional wound care.

The patient was also reviewed by the psychiatric team who made a diagnosis of schizophrenia and commenced the patient on haloperidol. He was to be followed up on an outpatient basis in the psychiatry clinic.

The patient reported normal nocturnal erections while on admission. Further evaluation of the suspected urethral injury with urethrogram and a urethroscopy was planned but this was declined by the patient who opted to retain his suprapubic catheter.

**Figure 5:**
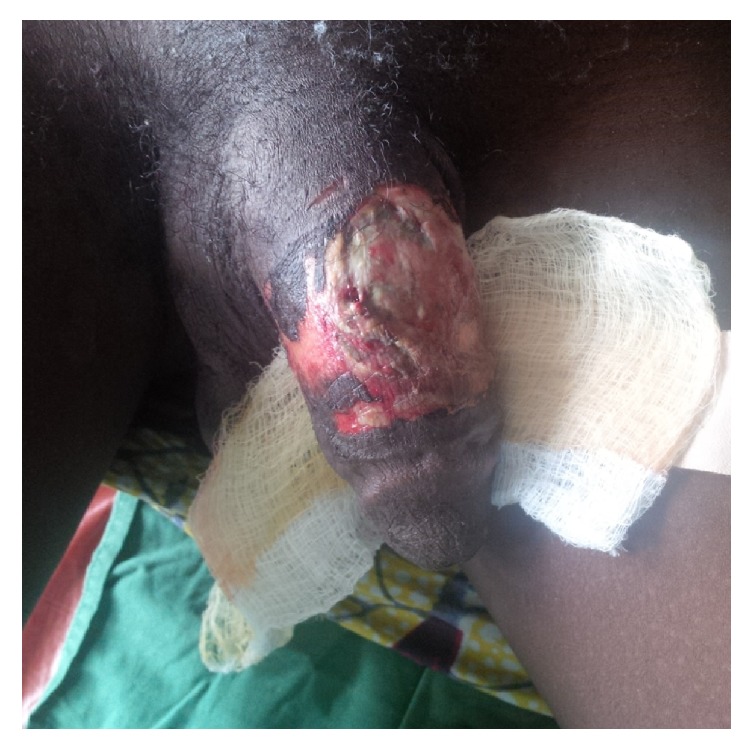
Penile ulcer granulating well 2 weeks after removing the constricting ring.

The patient also declined any additional wound care by the plastic surgery team and the wound was healing satisfactorily by secondary intention as at 2 weeks after the initial presentation (Figure 5).

He subsequently defaulted from care.

## 3. Discussion

The first published report of penile strangulation injury was by Gauthier in 1755 [12].

Since then there have other reports describing the use of penile constricting devices.

The use of penile constricting devices can lead to various degrees of vascular obstruction which can be as mild as venous constriction which resolves after decompression, to arterial occlusion which can lead to gangrene [13].

The motivation for the insertion of penile constricting devices varies. The most common motivation described in adults has been for erotic stimulation [6]. Other reasons have also been reported including control of enuresis and psychiatric abnormalities [14]. Our patient had a psychiatric illness and denied any sexual motivation.

Our patient's use of a metallic ring as a penile constricting device is in keeping with the report by Silberstein et al. that the most common device that results in penile entrapment is a metal ring [6].

Numerous methods have been described in the literature for the removal of penile constricting rings and these include lubrication of the shaft followed by a manual attempt at removal of the device, the string method, penile degloving, and use of industrial drills, steel saws, hacksaws, and high-speed electric drills [4, 7, 8]. Various factors influence the choice of the method of removal of the constricting device and these include the nature and thickness of the constricting device, surgeon's expertise or resourcefulness, extent of the penile injury, and the particular equipment available for use [14].

Irrespective of the aetiology, the use of a strangulating penile device is a urologic emergency which requires immediate decompression to guarantee a favorable outcome. According to the grading of constricting penile injuries by Bhat et al., our patient had grade III injury which included marked penile swelling distal to the constricting device, skin ulceration at the site of constriction, and reduced penile sensation with suspected urethral injury [11].

Initial attempts to remove the constricting ring using generous lubrication, corporal aspiration, and application of ice to decompress had failed. The very marked penile swelling precluded the use of the different methods of penile degloving. We were also unable to cut through the very thick constricting device with the manual saws available in the hospital operating theatre and the engineering department, hence a timely decision was made to call the Lagos State Fire Service whose personnel successfully cut the constricting device with a power driven saw in time to avoid penile gangrene.

It has been documented that manual cutting may sometimes be insufficient to cut through very thick steel material of industrial grade [1]. In such cases, it is imperative that an early decision is made to call for the appropriate cutting equipment from the necessary quarters and avoid delays that may inevitably lead to complications like penile gangrene and fistula formation amongst others. This is particularly important when penile gangrene might be imminent as in our patient.

The source of the cutting equipment may be immaterial considering the time constraint the urologist is sometimes faced with in some very severe cases of penile constriction injuries. McGain et al. had indeed documented the use of a portable glass cutter from a commercial glass supply retailer for cutting a penile constricting device [4]. The Lagos State Fire Service was the only source of an appropriate power driven saw available to us in the management of this index case.

Kore and Blacklock were the first to report the use of the Fire Service in the removal of a penile constricting device [10]. Since then, other workers have also described the successful removal of penile constricting devices with the assistance of the Fire Department [15–17].

There have been reports of strangulation penile injuries in Nigeria [2, 7, 8, 18]. Our report is however the first report of the help of the Fire Service in the removal of a penile constricting device in Nigeria.

The need to protect patients from thermal burns while using sawing and drilling equipment for the removal of constricting penile devices has been stressed by earlier workers [11, 19]. We achieved this by cooling the penis with ice packs and insinuating artery forceps between the penile skin and the constricting device while operating the powered saw. Hence the patient did not have any major additional penile injuries. When patients however have injuries in the course of removal of the constricting penile device, such injuries generally heal well [18].

The decent recovery made by the patient without any major damage to the penis justified the early distress call made for support from outside the hospital.

## 4. Conclusion

There is no standard treatment modality for the removal of penile constricting devices. Every case needs an individual approach depending on the circumstance and the armamentarium available to the attending surgeon.

Our report again brings to the fore the potential dangers of penile constricting devices and the need for early presentation and intervention as most of the easier techniques of removal of the devices are generally effective only when the patient presents early and the penis is not too swollen.

The management of this rare clinical entity therefore remains a challenge to the urologist and sometimes requires ingenuity for successful treatment. Achieving a timely and successful outcome may require a multidisciplinary approach involving equipment only available with the Fire Service or other emergency agencies.
